# Constructing a bilingual website with validated database for Herb and Western medicine interactions using Ginseng, Ginkgo and Dong Quai as examples

**DOI:** 10.1186/s12906-019-2731-1

**Published:** 2019-11-27

**Authors:** Chang-Shiann Wu, Yu-Huai Chen, Chi-Liang Chen, Sheng-Kan Chien, Nailis Syifa, Yu-Chun Hung, Kai-Jen Cheng, Shu-Chin Hu, Pei-Tzu Lo, Shun-Yung Lin, Tzu-Hua Wu

**Affiliations:** 1 Department of Information Management, College of Management, National Formosa University, No. 64, Wunhua Rd, Huwei Township, Yunlin County 632 Taiwan; 2 Division of Clinical Pharmacy, School of Pharmacy, College of Pharmacy, Taipei Medical University, 250 Wu-Hsing Street, Taipei 110, Taiwan; 3 Department of Pharmacy, Taipei Veterans General Hospital, 201 Sec II, Shipai Rd., Beitou Dist, Taipei City 112, Taiwan; 4 Department of Pharmacy, Keelung Municipal Hospital, No. 6, Ln. 2, Yi 2nd Rd., Zhongzheng Dist, Keelung City 202, Taiwan; 5 Pharmacy Department, Faculty of Health Science, University of Muhammadiyah Malang, Jl. Raya Tlogomas No. 246, Malang 651, Indonesia; 6 Psychiatric Research Center, Wan Fang Hospital, Taipei Medical University, No. 111, Section 3, Xing-Long Road, Taipei 116, Taiwan

**Keywords:** Bilingual website, Herb-drug interaction, Database, Reliability, Smart search

## Abstract

**Background:**

Concerns have been raised regarding the efficacy and safety resulting from the potential interactions of herbs with Western medications due to the use of both herbs and Western medicine by the general public. Information obtained from the web must be critically evaluated prior to its use in making decisions.

**Description:**

This study aimed to construct an herb-drug interaction (HDI) website (https://drug-herb-interaction.netlify.com) with a critically reviewed database. Node.js was used to store the database by running JavaScript. Vue.js is a front-end framework used for web interface development. A total of 135 sets of information related to the interactions of ginseng, ginkgo and dong quai with Western medicine from the literature identified in Medline were collected, followed by critical reviews to prepare nineteen items of information for each HDI monograph. A total of 80 sets of validated HDIs met all criteria and were further assessed at the individual reliability level (likely, possible, and unevaluable) and labeled with the “interaction” item. This query system of the website can be operated in both the Chinese and English languages to obtain all monographs on HDIs in the database, including bilingual interaction data. The database of HDI monographs can be updated by simply uploading a new version of the information Excel file. The designed “smart search” module, in addition to the “single search”, is convenient for requesting multiple searches. Among the “likely” interactions (*n* = 26), 50% show negative HDIs. Ten of these can increase the effect of the Western drug, and the others (*n* = 3) imply that the HDI can be beneficial.

**Conclusions:**

The current study provides a website platform and 80 sets of validated bilingual HDIs involving ginseng, ginkgo and dong quai in an online database. A search of HDI monographs related to these three herbs can be performed with this bilingual, easy-to-use query website, which is feasible for professionals and the general public. The identified reliability level for each HDI may assist readers’ decisions regarding whether taking Western medications concomitant with one of three herbal medicinal foods is safe or whether caution is required due to potentially serious outcomes.

## Background

Patients have increasingly turned to the Internet for health-related information since the end of the twentieth century [1]. Evidence suggests that healthcare consumers have become more proactively involved in the management of their own health due to the large volume of information available. During the 10 years of the Web 1.0 era, general Internet users were simply receivers of content and could only browse content created by certain companies and published on websites by webmasters. During the Web 2.0 era [2], Internet usage has changed. Users can share their own written articles, photos, and videos online, and blogs, online photo albums, and video sharing have become popular on the Internet. Furthermore, users can use the Internet to quickly catch up with friends and interact with them through mini online games. These types of social websites have emerged rapidly and consecutively, and the contents of those online information can be misleading since it was published without a peer-reviewed process. Therefore, specific websites presenting literature on herb-drug interactions (HDIs) summarized by professionals with pharmacy and/or medical research backgrounds are needed to prevent readers from using informational content inappropriately.

Vast amounts of information regarding Western medicines is available, and the current medical information on websites in Taiwan is quite open and accessible. Because many people receive prescriptions for Chinese or Western medicines in different clinical settings, health professionals, including physicians and pharmacists, may not be aware that Chinese and Western medicines are consumed by patients at the same time. A study utilizing the National Health Insurance Database of Taiwan [3] showed that in 2007, a high percentage of Western medicine users also used Chinese medicine. This prevalence rate does not include people who purchase complementary herbal medicines independently.

To date, available information regarding HDIs in Taiwan is limited. Most resources only provide certain information scattered on different websites. Interactions between herbs and Western medicine can cause undesirable effects, but these medications may also work together to enhance treatment efficiency while reducing side effects. The importance of considering the interaction effects between herbs and Western medicine cannot be overemphasized and has been mentioned in previous studies [4]. Therefore, the quality and empirical validity of information regarding interactions between herbs and Western medication [5] and communication between medical professionals and the general public are highly important. However, very few studies [5–7] have assessed the quality of online HDI information, and none of these studies have evaluated sites intended for the consumer. In addition, general open access websites have various shortcomings [8] because most of the information does not have to strictly reference the literature and relate to clinical applications [9]. As the volume of PubMed records increases, it is necessary to prepare a systematic database. The query method used on these websites mostly shows only one item at a time and cannot simultaneously display multiple items. The main objective of this study is to create an easy-to-use search website with validated HDI database for use by physicians, pharmacists, and the general public.

## Construction and content

### Source of information

To prepare an example of a bilingual website with validated information, a systematic search for HDI information limited to three commonly used herbs was retrieved from Medline which is an academic literature database. In this study, HDI information content from original studies which cited in “review type” articles was extracted into the current database. The process regarding the collection of “review type” articles is described as follows:

First, two senior researchers who had more than twenty years of experience in information management or clinical research fields performed literature searches together in the Medline database via the PubMed® website (http://etds.ncbi.nlm.nih.gov/pubmed/) using the following terms: “Herb–Drug Interactions”[Mesh] OR “Food–Drug Interactions”[Mesh]) OR “Chinese medicine”[Mesh]) OR “Dietary Supplements”[Mesh]) OR “Herb Extracts”[Mesh])) as described in a recent drug-drug interaction study [10] and limited to “review type” articles. The purpose of the selected “review” articles was to provide systematic descriptions of herbal and Western medicine interactions.

Second, the identified review articles were collected if met the following criteria: 1) abstract content was related to the keywords “ginseng or *Panax ginseng* ginsenoside”, “*Ginkgo biloba*”, and “danggui or dong quai or *Angelicae sinensis*”; 2) publication years were during the period of 1999–2018; 3) The full text of each identified review article was downloaded and read thoroughly by at least two senior pharmacists to search for interaction information for three herbs, including their reference lists. This required a tremendous amount of reading but avoided the exclusion of literature on herb-drug interaction topics. Articles that did not describe Western drug interactions with any of the three herbs were excluded. Because limiting the keywords by MeSH constraints may reduce the number of studies returned, searches were also executed without constraints by using the keywords “ginseng or *Panax ginseng* ginsenoside”, “*Ginkgo biloba*”, and “danggui or dong quai or *Angelicae sinensis*” to retrieve additional review articles on HDIs to fulfill the additional criterion that at least one “systematic review article” every other year was retrieved to minimize the possibility of missing information. A total of fourteen “review” articles [11–24] published during the prior twenty years were collected.

### Information extraction and rewording

HDI information from the original studies cited in the review articles related to ginseng, ginkgo and dong quai, commonly interacting herbs [25], was included for information extraction to the current database. In the fourteen retrieved review articles, 120 sets of HDI information were mentioned in original articles or case reports. The doses of the herb-drug combinations were also recorded in relation to “patient situation” or “study design” to allow professionals to identify the appropriate doses when applying the website information in consultations. The human studies included case reports, case series, randomized or nonrandomized clinical trials and other types of studies. Literature information was extracted, such as the titles, authors, periodicals, sources, and hyperlinks of references for each monograph, and the evidence level of the study design was classified as an in vitro or in vivo animal experiment, clinical trial, or patient case. The advantage of this database is that it includes negative results of interactions to demonstrate their safety information.

After two junior pharmacists read the report contents, 55 sets of HDI were excluded due to repeated information, information on herb-herb interactions, a lack of dose information, unavailable original content or evidence that was not clearly described. The specific pairs of herb-drug interactions that were excluded due to unclear descriptions of information were then searched again on PubMed without restricting the MeSH topic by using the specific pairs of herb-drug names to retrieve the original articles that reported their interactions since MeSH for Medline is updated once a year. Fifteen additional “original” types of articles, mostly newly published randomized controlled trials, were collected, and their information was extracted and reworded for each of the HDI studies as described previously. Concise information in English on nineteen items (Table 1) for each HDI monograph was extracted by rewriting the content concisely (word count<30) according to the original study content. Interaction information regarding the interaction results, study design, mechanism or recommendations was also provided in condensed Chinese comparable with the website environment. Bilingual HDI information met the needs or preferences of domestic and foreign users. Eighty sets of HDIs from 70 references related to ginseng, ginkgo, and dong quai were available for this website.

**Table 1:**
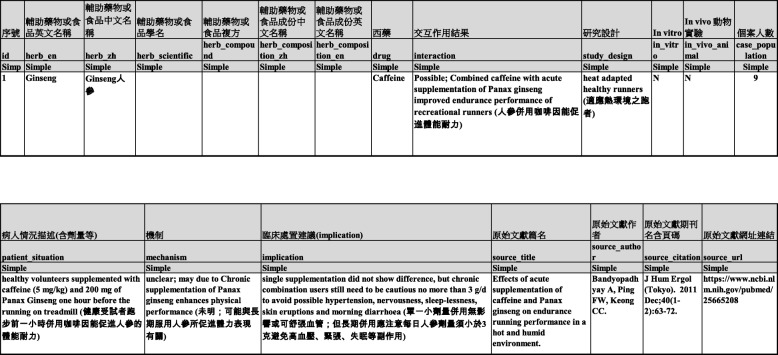
Columns of Json database items and one example of bi-lingual information content for ginseng interaction

### Validating the content of HDI monographs

To validate the content of nineteen items for each HDI monograph, first, information related to endpoint changes, such as drug concentration or efficacy (e.g., symptoms, biochemical lab data) was identified from the respective reference.

Second, at least two senior registered pharmacists (with more than 10 years of experience) or professors of clinical pharmacy read each of the collected articles to reconfirm the study design or how the drug interactions occurred. All recommendations for managing the listed HDIs were finalized by consensus reached via discussion by two senior pharmacists. If consensus was not reached, a professor of clinical pharmacy finalized the extracted information of each item for the monographs including its reliability score. Report reliability for all reported HDI content and data was also assessed according to the reliability scale adopted from a previous study [26], and the clinical relevance was critically categorized into three levels of reliability (unevaluable, possible or likely). The lowest level was not clinically relevant, the second was possible, and the highest reliability interaction was considered potentially clinically relevant. If the reliability scores from the two pharmacists did not reach agreement, the score was further judged by a professor of clinical pharmacy according to the article content and considerations of patient safety. Among 80 sets of HDI monographs (as shown in Additional file 1: Table S1), 30 sets (9 sets for ginseng and 21 sets for ginkgo) were identified as “likely” HDIs, which could be potentially clinically relevant. Eleven sets of HDIs were identified as “unevaluable”, which means the report was not conducted with humans (*n* = 10) or contained inadequate information to assess the likelihood of an interaction. The rest of the monographs were identified as “possible” interactions (*n* = 39 sets) due to those reports provided some evidence of an interaction, but there may have been other causes of the event. Seven possible HDIs reported increased risks with a reliability score close to the margin of the “likely” category. Those HDIs were mostly related to increased bleeding risks with ginseng/ginkgo or lower tolbutamide levels or CNS drug effects with ginkgo. Future work is needed to clarify these issues.

### The HDI web design

**Fig. 1:**
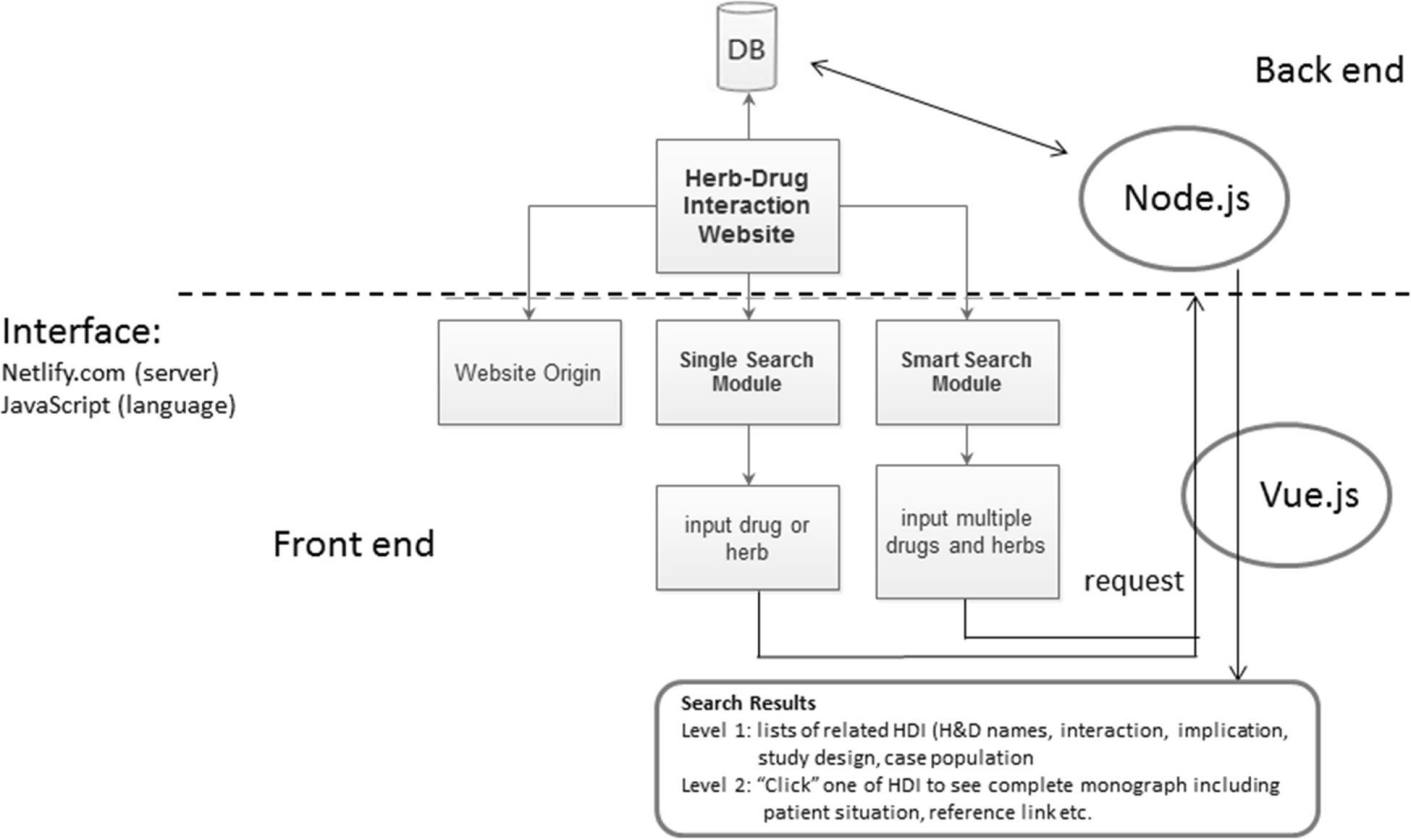
The functional framework for the current HDI web design

Web development technology has undergone many evolutions, and current technologies require JavaScript to operate. To simplify JavaScript development and improve web developers’ productivity, jQuery is undoubtedly among the most popular JavaScript libraries in recent years. Furthermore, many developers have used JavaScript for server-side applications [27]. Because Node.js is a JavaScript library used on the server side of Netlify (GitHub, Inc) [28], this study constructed an HDI website (https://drug-herb-interaction.netlify.com) employing Node.js to store the critically reviewed database and Vue.js, a front-end framework, to run data searching via the web interface. The functional framework for current HDI web design is summarized in Fig. 1. The vue-i18n suite is used to allow both Chinese- and English-speaking users to understand the system interface. The details regarding how to implement the system, establish an online database, and design web pages are described in the Additional file 2.

### Main page

**Fig. 2:**
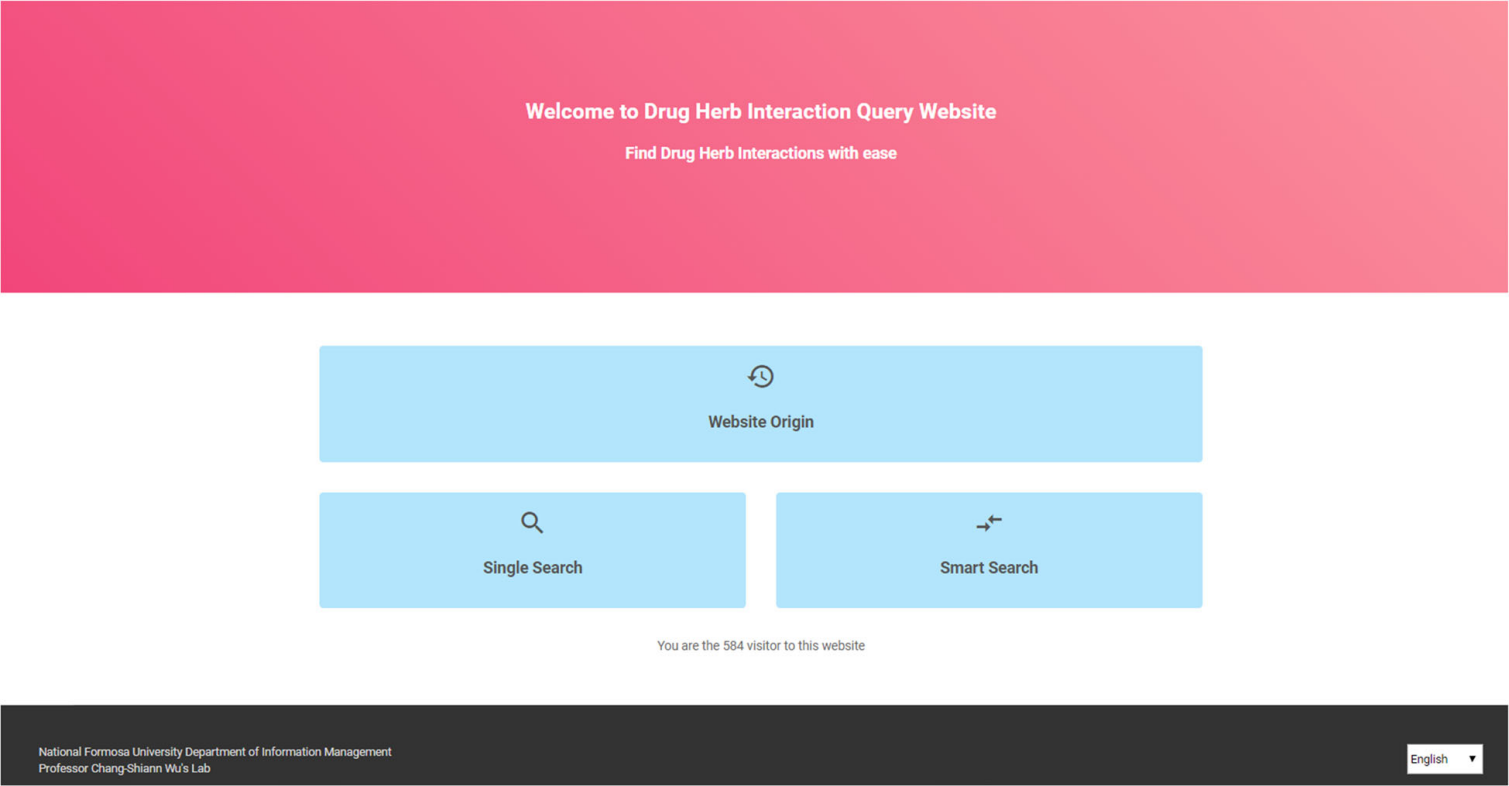
Homepage of HDI website

The main page of the website (https://drug-herb-interaction.netlify.com/) is shown in Fig. 2. The main page can detect the user’s language. There is a “selector” button in the website’s footer at the lower right corner that allows users to select their desired language. The website origin, search options and total number of visitors are displayed in the center of the page. After a single or smart search has been chosen, the user can directly enter keywords on the search screen. The total number of searches is also displayed on the page.

### Utility and search screen

There are three search methods, as follows: 1. the herb name is inputted and searched; 2. the Western medicine name is inputted and searched; and 3. the herb and Western medicine names are inputted and cross-searched. The page also allows users to perform fuzzy searches and fuzzy cross-searches. Users who do not know the entire name of a medicine can enter one of the characters in the name to search and/or cross-search. This study prepared HDI information monographs consisted of nineteen items unless the information was not available. The search results list the herb names (Chinese, English, and scientific names), herb prescriptions, Western medicine names, ways the medicines can interact, interactions, implications or recommendations, patient situation or study design, and original article names and origins.

The search screen includes the following two fields: herb and Western medicine. The herb field accepts Chinese herb names, English herb names, scientific herb names and herb prescriptions. The Western medicine field accepts Western medicine names. For single searches, the user’s keyword is transmitted directly to the fuse to initiate the search. Then, all search results appear directly on the screen.

**Fig. 3:**
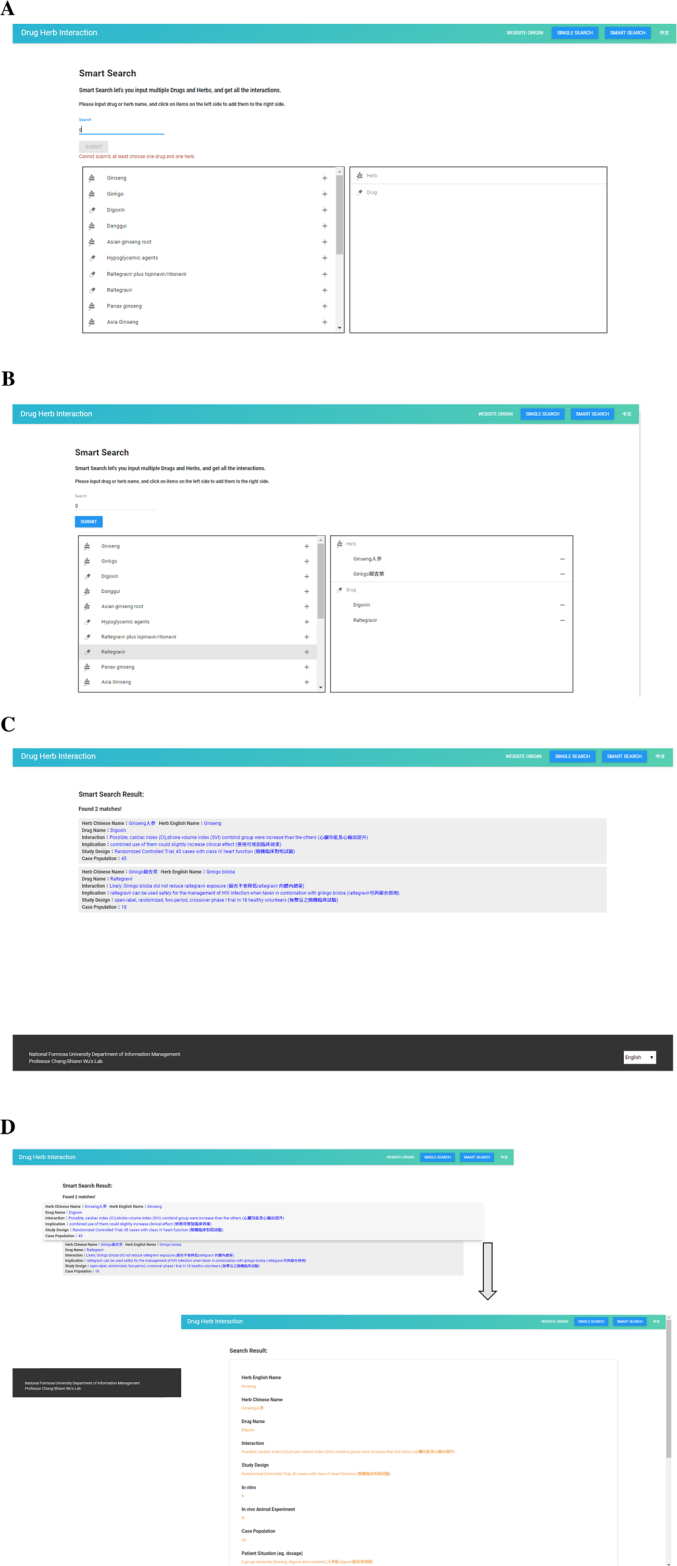
**a** Smart search webpage. **b** One English letter “g” is input, and users can select the item of interest by clicking “+” to the right column; users can continuously click “+” for multiple selections of herbs and drugs, followed by clicking the “submit” icon to command the query. **c** Display of matched results lists. **d** Pointing out the HDI text box and clicking to pop out the selected monograph information for each item

Moreover, the website implements “smart search” functions, which were also applied on Micromedex® to search drug information. Users need to input both the herb name and the Western medicine name (selecting at least one herb name into the right column by clicking “+” as a cross-search candidate, completing a similar action for the Western medicine name, then clicking “Submit”) to start a cross-search for a further interaction query. Only sets of HDI information matches for both selected herbs and drugs are displayed on the screen. Figure 3a shows that the user can key in only one letter to obtain quick search results without needing to key in the full name of the herb or drug. Example results of a smart search inquiry by selecting two herbs and two drugs from the listed matches for the letter “g” are shown in Fig. 3b. Figure 3c displays the above cross-searches, and Fig. 3d shows how to view all information on each item.

After the search, the first output shows the eight items of the matched HDI monograph. If the user is interested in obtaining more detailed information, such as the patient situation or reference link, all information on nineteen items for each monograph can be displayed by clicking the icon of the HDI from the matched results.

### Website database maintenance

Given the continuous updating of information regarding interactions between herbs and Western medicines, errors are possible when nontechnical personnel update their data in accordance with the JSON format. In our system, the Apps Script function in Google Drive can be used to authorize database updates. Google Drive’s management system with comprehensive rights provides the ability to allow multiple people to edit the same document simultaneously. Additionally, many of Google Drive’s existing features can be used directly. Because many people are already familiar with this tool, our system is easier to manage. When connecting the website settings center to the back-end database, users can customize the search and display settings in addition to the original materials regarding the interactions between herbs and Western medicines. After modifying the data, the selected Publish button created by the Apps Script in Google Spreadsheet is pressed to package the new data into the JSON format and upload it to the cloud to complete the update.

## Discussion

This website is the first to provide quick searching for relevant and concise bilingual HDI information because Vue.js provides a static front-end framework for the web interface, which is convenient for readers fluent in English and those who are not. The current HDI information does not adopt English paragraphs directly from the literature; instead, it rewords the respective content to be more concise and then becomes understandable Chinese wording after translation. Word counts are also limited, so readers can easily grasp the knowledge value through improved readability (82.4% vs. 96.2%; data not shown). To provide validated information, in addition to interaction content extracted from references collected in the database, the current database information includes the reliability level by assessing each set of study content. This website, which has a smart search function, was built by a multidisciplinary team that included both computer information engineers and pharmacists to provide the HDI study design and detailed results with quantitative information. Given these strengths, pharmacists and consumers can use this website to quickly search for possible interactions and determine whether using the medicines together could pose health risks or provide additional benefits. When providing consultations, pharmacists may be able to critically facilitate clearer decisions for patients. Consumers may also want to actively consult with their health professionals before starting or stopping any medications, including Chinese herbal medicines. More than 25% of residents in Taiwan use both Chinese and Western medicines [29]. Furthermore, elderly patients aged > 65 years and those with Alzheimer’s disease [30] are usually prescribed more than three different medicines, and progressive impairment in the functional reserve of multiple organs may affect pharmacokinetics or pharmacodynamics during aging [31]. Therefore, the vulnerability of the elderly to problems related to drug use is increasing [32]. Professionals could regularly educate their patients using this HDI web information before recommending herbal medications.

In terms of the system construction, this research is based on Node.js using Google’s V8 engine. This work applies JavaScript to a native machine code and executes quickly. Node.js uses event-driven non-I/O blocking models retrieved on 2017/11/22 from https://github.com/nodejs/node/wiki) [28]. Thus, this work can deliver lightweight, efficient performance in data-hungry, real-time Web applications and significantly shorten search times. The syntax supported by IE is relatively old, at least in JavaScript terms. Hence, the recommended environment is to use the latest Chrome/Firefox/Safari rather than IE. Google Drive CMS is a convenient tool for health professionals to update HDI information.

According to our database, although a limited number (a total of 135 sets of HDIs, including 120 from review articles and 15 from recent original articles) of potential pharmacokinetic and/or pharmacodynamic HDIs have been examined and critically assessed, only 6 sets of HDIs related to ginseng and 20 sets of HDIs related to ginkgo out of 80 sets of information were considered “likely”. Among these “likely” interactions (*n* = 26), 50% showed negative HDI results, which is supported by a recent review on HDIs with warfarin indicating that the incidence of adverse events in all trials was low and there were no major adverse events [33]. Some HDIs increased the effect of the Western drug, whereas others (*n* = 3) implied that the HDI could be beneficial.

Among the most reliable sets of HDIs, two HDI results for ginseng show negative interactions (no significant change when combined with warfarin), but one set of HDI results indicates an increased risk of blood clots when combined with warfarin, and the other three suggested that this combination might enhance the efficacy of antihypertensive medication or antagonize the effects of diuretics. Eleven sets of HDI results for ginkgo were negative interactions (no significant change when combined with various Western drugs, such as caffeine, dextromethorphan, diclofenac, fexofenadine, lopinavir/ritonavir, omeprazole, raltegravir, SSRIs, talinolol, tolbutamide, and warfarin. The other nine sets of HDI results were recognized to cause significant alteration of response to modern medicines (5-fluorouracil, alprazolam, haloperidol × 2, metformin, midazolam, nifedipine × 2, talinolol). The current database reports that four of the CNS drugs that “likely” interacted with ginkgo are different from those in a previous report in which HDI risks were presented only for grapefruit and St. John’s wort but not for ginseng or ginkgo [34] in patients with neurological disorders. In fact, the HDI information shows two beneficial effects [35]: ginkgo combined with haloperidol for disease improvement and reduced sedative medication exposure. In addition, ginkgo tends to increase blood concentrations of antihypertensive drugs at higher doses and improve glycemic control and the chemotherapy side effects.

The current platform provides database information for nineteen different items for each monograph, including herb-drug names, interaction results, integrated information and levels of reliability in an effort to improve quality. Three of the chosen herbs are classified as medicinal foods in Taiwan, and people may use them as daily supplements. Taiwan currently has two websites that address the interaction between herbs and Western medicine. 1) The Chimei search system (http://www.chimei.org.tw/main/cmh_department/55500/DIS/cdrug_interaction1.asp) is a website built by pharmacists working at a hospital to provide search functions for both hospital staff and outside users. Users can input Chinese in the herb field and scientific names and product names (in both Chinese and English) in the Western medicine field. However, information was limited to those items available in the hospital and the source of the original documents is unknown. (2) The Chinese-Western medicine integrative information network (http://dhi.cmu.edu.tw/query/) was created in 2004 by the Dept. of Health’s Traditional Chinese Medicine Committee as a part of their “Five-Year Chinese Medicine Safety Plan” managed by China Medical University (CMU). Currently, the CMU webpage design is easy to operate. More than one traditional Chinese medicine and Western medicine name can be input simultaneously for querying. However, their query output only shows a brief description of the interaction without detailed study information. In addition, the displayed Chinese herbal name does not show the origins or scientific name. Clicking the reference link shown on the result webpage links to the abstract content on PubMed, in contrast to the database introduced here, which provides extracted information for specific items. Moreover, most of the literature linked by the CMU’s website is not open access. Therefore, the general population may not be able to download the full text if a detailed patient situation is needed.

Even though the database is small and general techniques of system implementation were utilized to construct the current website environment, it provides an example of easy website maintenance by pharmacists. The advantage of this website system is its ability to search quickly, which may be critical to attract the general population because they may not be able to correctly key in complete herb or drug names. It is also beneficial for healthcare professionals who need to make a decision or diagnose within 1 minute whether a patient’s problem is due to the risk of HDI. Our future goal is to expand the database as a drug information service. The current HDI website was also designed to provide a smart search environment for pharmacists who usually use commercial health information databases that can minimize Internet technology maintenance loads and improve the quality of information and satisfaction for users.

Research conducted abroad in the UK [36] and the US [37] has reported that at least 15 million people are at risk of a harmful interactions between prescription medications and herbal/vitamin supplements [38]. This is particularly dangerous because many people do not take the time to tell their doctors about the herbal/vitamin supplements they are taking [39]. The most important issue is whether medical professionals, including pharmacists [40], take the initiative and openly communicate with patients about their usage of Chinese and Western medicines [41]. This study provides a communication and education tool. The functions built into this study’s website include bilingual information and the ability to simultaneously input multiple Chinese/Western medicines in one search with a smart search and obtain results related to drugs that may create interactions for practical application.

This study has certain limitations. For example, the current version of the database covers only information related to ginseng, ginkgo and dong quai. Because small populations possibly as low as 1 % exclusively use alternative medicine in Greek [42], more HDIs of the other herbal medicines need to be included, especially for patients with a variety of debilitating neurological and psychiatric disorders [34] or for the elderly who use alternative medicine, including Chinese medicine, and who potentially also use Western medicine [43]. The Chinese information content, including medical terms, can be further improved via the readability assessment tool to make it more easily understood by the general public because the content is written by professionals with medical backgrounds who are not experts in language communications. Whether the concomitant use of herbs and Western medication presents a real problem or a benefit in practice must be identified through individualized care [44].

## Conclusions

The website in this study includes an embedded 80-set HDI information database on ginseng, ginkgo and dong quai with a smart search function built by a multidisciplinary team including both information management specialists and pharmacists. Using the highly efficient, easy-to-expand Node.js, JavaScript can allow pharmacists to execute database expansion on the same server platform without the assistance of computer technicians. By using this website, healthcare professionals can provide HDI information linked to the academic literature, especially for patients taking these medications with prescription medications. Although only one-third of the literature on HDIs in the current database was clinically relevant, an open-minded approach during patients’ visits is to counsel patients with enough information about the signs and symptoms of herb-drug interactions to allow patients to recognize whether the combined use of herbs and drugs may be safe or if an adverse event may occur. This work created an easy-to-use HDI query website in both English and Chinese for use by physicians, pharmacists and the general public, including people who can only read Chinese.

## Supplementary information

**Additional file 1:** Table for HDIs.

**Additional file 2:** Supplementary Information.

## Data Availability

Not applicable.

## Abbreviations

CMU: China Medical University
HDI: Herb Drug Interaction
JSON: JavaScript Object Notation
MVVM: Model-View-ViewModel

## References

[1] Cline RJ, Haynes KM (2001). Consumer health information seeking on the internet: the state of the art. Health Educ Res.

[2] Mohammed S, Orabi A, Fiaidhi J, Orabi M, Benlamri R (2008). Developing a web 2.0 telemedical education system: the AJAX-cocoon portal. Int J Electron Healthc.

[3] Chen MC, Lai JN, Chen PC, Wang JD (2013). Concurrent use of conventional drugs with Chinese herbal products in Taiwan: a population-based study. J Tradit Complement Med.

[4] Lin S: The construction of websites that provide info on interactions between Western and Chinese medicines. In: China Medical University. Taiwan; 2005.

[5] Adam TJ, Vang J (2015). Content and usability evaluation of patient oriented drug-drug interaction websites. AMIA Annu Symp Proc.

[6] Scarton LA, Del Fiol G, Treitler-Zeng Q (2013). Completeness, accuracy, and presentation of information on interactions between prescription drugs and alternative medicines: an internet review. Studies in health technology and informatics.

[7] Wong FW, Lin L, Lim DC (2009). Drug and herb interactions: searching the web. Aust Fam Physician.

[8] Raban MZ, Tariq A, Richardson L, Byrne M, Robinson M, Li L, Westbrook JI, Baysari MT (2016). Evaluation of web-based consumer medication information: content and usability of 4 Australian websites. Interact J Med Res.

[9] Taveira-Gomes T, Ferreira P, Taveira-Gomes I, Severo M, Ferreira MA (2016). What are we looking for in computer-based learning interventions in medical education? A systematic review. J Med Internet Res.

[10] Lu Y, Figler B, Huang H, Tu YC, Wang J, Cheng F (2017). Characterization of the mechanism of drug-drug interactions from PubMed using MeSH terms. PLoS One.

[11] Cupp MJ (1999). Herbal remedies: adverse effects and drug interactions. Am Fam Physician.

[12] Cheng TO (2000). Herbal interactions with cardiac drugs. Arch Intern Med.

[13] Rogers EA, Gough JE, Brewer KL (2001). Are emergency department patients at risk for herb-drug interactions?. Acad Emerg Med Off J Soc Acad Emerg Med.

[14] Kuhn MA: Herbal remedies: drug-herb interactions. Crit Care Nurse 2002, 22(2):22-28, 30, 32; quiz 34-25. 11961942

[15] Kiefer D, Pantuso T (2003). Panax ginseng. Am Fam Physician.

[16] Cheng KF, Leung KS, Leung PC (2003). Interactions between modern and Chinese medicinal drugs: a general review. The American journal of Chinese medicine.

[17] Brazier NC, Levine MA (2003). Understanding drug-herb interactions. Pharmacoepidemiol Drug Saf.

[18] Chavez ML, Jordan MA, Chavez PI (2006). Evidence-based drug--herbal interactions. Life Sci.

[19] Ulbricht C, Chao W, Costa D, Rusie-Seamon E, Weissner W, Woods J (2008). Clinical evidence of herb-drug interactions: a systematic review by the natural standard research collaboration. Curr Drug Metab.

[20] Chen XW, Sneed KB, Pan SY, Cao C, Kanwar JR, Chew H, Zhou SF (2012). Herb-drug interactions and mechanistic and clinical considerations. Curr Drug Metab.

[21] Izzo AA (2012). Interactions between herbs and conventional drugs: overview of the clinical data. Medical principles and practice : international journal of the Kuwait University, Health Science Centre.

[22] Choi JG, Eom SM, Kim J, Kim SH, Huh E, Kim H, Lee Y, Lee H, Oh MS (2016). A comprehensive review of recent studies on herb-drug interaction: a focus on Pharmacodynamic interaction. J Altern Complement Med.

[23] Awortwe C, Makiwane M, Reuter H, Muller C, Louw J, Rosenkranz B (2018). Critical evaluation of causality assessment of herb-drug interactions in patients. Br J Clin Pharmacol.

[24] Jalloh MA, Gregory PJ, Hein D, Risoldi Cochrane Z, Rodriguez A (2017). Dietary supplement interactions with antiretrovirals: a systematic review. Int J STD AIDS.

[25] Chen KC, Lu R, Iqbal U, Hsu KC, Chen BL, Nguyen PA, Yang HC, Huang CW, Li YC, Jian WS (2015). Interactions between traditional Chinese medicine and western drugs in Taiwan: a population-based study. Comput Methods Prog Biomed.

[26] Fugh-Berman A, Ernst E (2001). Herb-drug interactions: review and assessment of report reliability. Br J Clin Pharmacol.

[27] O’Halloran DM (2017). Phylo-node: A molecular phylogenetic toolkit using Node.js. PLoS One.

[28] Node.js community wiki [https://github.com/nodejs/node/wiki © 2017 GitHub Inc.]

[29] Chao LP: Survey of Concurrent Use of Chinese Medicine and Western Medicine in Cental Taiwan, vol. 21; 2003.

[30] Lai CY, Wu MY, Chiang JH, Sun MF, Chen YH, Chang CT, Lin JG, Yen HR (2017). Utilization of Western medicine and traditional Chinese medicine among patients with Alzheimer’s disease in Taiwan: a nationwide population-based study. Eur J Neurol.

[31] Corsonello A, Abbatecola AM, Fusco S, Luciani F, Marino A, Catalano S, Maggio MG, Lattanzio F (2015). The impact of drug interactions and polypharmacy on antimicrobial therapy in the elderly. Clin Microbiol Infect.

[32] Secoli SR (2010). [Polypharmacy: interaction and adverse reactions in the use of drugs by elderly people]. Polifarmacia: interacoes e reacoes adversas no uso de medicamentos por idosos. Rev Bras Enferm.

[33] Choi S, Oh D-S, Jerng UM (2017). A systematic review of the pharmacokinetic and pharmacodynamic interactions of herbal medicine with warfarin. PLoS One.

[34] Wilson V, Maulik SK (2018). Herb-drug interactions in neurological disorders: a critical appraisal. Curr Drug Metab.

[35] Singh A, Zhao K (2017). Herb-drug interactions of commonly used Chinese medicinal herbs. Int Rev Neurobiol.

[36] Posadzki P, Watson LK, Alotaibi A, Ernst E (2013). Prevalence of use of complementary and alternative medicine (CAM) by patients/consumers in the UK: systematic review of surveys. Clinical medicine.

[37] Fox S, Rainie L, Horrigan J, Lenhart A, Spooner T, Carter C. Trust and privacy online: why Americans want to rewrite the rules. The Pew Internet & American Life Project. 2000:1–29.

[38] Smolinske SC (1999). Dietary supplement-drug interactions. J Am Med Wom Assoc.

[39] Asher GN, Corbett AH, Hawke RL (2017). Common herbal dietary supplement-drug interactions. Am Fam Physician.

[40] Klepser TB, Klepser ME (1999). Unsafe and potentially safe herbal therapies. Am J Health Syst Pharm.

[41] Shelley BM, Sussman AL, Williams RL, Segal AR, Crabtree BF, Rios net C (2009). ‘They don’t ask me so I don't tell them': patient-clinician communication about traditional, complementary, and alternative medicine. Ann Fam Med.

[42] Sommer JH, Burgi M, Theiss R (1999). A randomized experiment of the effects of including alternative medicine in the mandatory benefit package of health insurance funds in Switzerland. Complementary therapies in medicine.

[43] Ni H, Simile C, Hardy AM (2002). Utilization of complementary and alternative medicine by United States adults: results from the 1999 national health interview survey. Med Care.

[44] Spanakis M, Sfakianakis S, Sakkalis V, Spanakis EG (2019). PharmActa: Empowering Patients to Avoid Clinical Significant Drug-Herb Interactions. Medicines.

