# Supplementary material for: Constructing a bilingual website with validated database for Herb and Western medicine interactions using Ginseng, Ginkgo and Dong Quai as examples
Source: BMC Complement Altern Med. 2019 Nov 27;19:335. doi: 10.1186/s12906-019-2731-1 (PMC6881993; doi:10.1186/s12906-019-2731-1)
Supplement: Supplementary file 2 — Additional file 2: Supplementary Information. [file 12906_2019_2731_MOESM2_ESM.docx]

**Supplementary Information**

System Implementation

First, node.js was downloaded and installed to add the node and npm commands. Npm is the node suite administrator. For example, npm can be used to install fuse.js for the fuse.js suite[[1](#_ENREF_1), [2](#_ENREF_2)] , as mentioned in the book Node.js Domain (v9.2.0. 2015). The folder was downloaded and extracted. The folder contains all the codes and README.md, which describes the instructions. The cd is used to switch to the folder with the materials; then, the npm install is executed to read package.json and install all suites required for the website. To continue editing on the local terminal after the website is created, npm run dev is executed to activate the server on the local terminal, and http://localhost:8080 can be opened in the browser. Additionally, while in developer mode, the webpack-dev-server can be used to save a file after making any changes. The program will be edited automatically, and the content displayed on the localhost will be updated. Hot Module Replacements or Live Reloads are very convenient and effective. The website’s search function uses the fuse.js suite. After the data are read by AJAX, the data can be directly fed to the new Fuse entity. Subsequent searches can be performed via fuse.search (keyword).

After the development is complete, git push can be used to push the content to GitHub, and Netlify’s deployment program will automatically be triggered. Then, the content will be compiled and uploaded onto the Netlify CDN platform to complete the automatic deployment process [[3](#_ENREF_3)].

Establishing an Online Database

The search function of the website was implemented using fuse.js, and the required formatting of the data was performed using JSON. Fuse.js is a lightweight JavaScript library which allows you to create a search box on the client-side to search moderately large data sets in a fuzzy fashion. The degree of fuzziness is set using a threshold from 0.0 to 1.0 whereby 0.0 requires a perfect match and a threshold of 1.0 would match anything. The JSON file is loaded via AJAX, and passed to a new Fuse instance. The .search() method supports the searching of simple arrays to deep key matching within complex objects. The results are sorted by score using a full Bitap algorithm, leveraging a modified version of the Diff, Match & Patch tool by Google.

JSON is short for JavaScript Object Notation and is a very lightweight data exchange format. JSON is based on the JavaScript Object Syntax format and was popularized by Douglas Crockford. Although JSON is based on JavaScript Object Syntax, it can be used independently. JSON can be read, analyzed, and generated in many programming environments. JSON is basically a file with a .json extension that has a MIME type of application/json. Its data types are strings, numbers, arrays, and objects.

JSON mainly uses a pair of curly brackets ({}) to enclose objects, a pair of square brackets ([]) to enclose arrays, and a pair of double quotation marks (””) to enclose strings. The variables are separated by commas, key-value pairs are used in the fields, and the contents can be in both English and Chinese. The sets of the interaction database were adopted from the individual articles, and all selected interaction information was compiled in a standardized form that was managed using Excel® spreadsheets. An example of HDI information is shown in Figure 1. The contents were re-written in English and Chinese to contain the most important information to make it suitable as a database for the website query. After the contents of all HDI monographs are pasted in the spreadsheet to replace the old data, website managers need to “click the “Google Drive CMS” to hit “Publish” to complete the database updating. The nineteen items of the HDI monograph are shown in Table 1.

| herb_en | 中藥英文名稱 | Herb English Name |
| --- | --- | --- |
| herb_zh | 中藥中文名稱 | Herb Chinese Name |
| herb_scientific | 中藥學名 | Herb Scientific Name |
| herb_compound | 中藥複方 | Herb Compound |
| herb_composition_zh | 中藥成份中文名稱 | Herb Composition Chinese |
| herb_composition_en | 中藥成份英文名稱 | Herb Composition English |
| Drug | 西藥 | Drug Name |
| interaction | 交互作用結果 | Interaction |
| study_design | 研究設計 | Study Design |
| in_vitro | In vitro | In Vitro |
| in_vivo_animal | In vivo 動物實驗 | In Vivo Animal Experiment |
| case_population | 個案人數 | Case Population |
| patient_situation | 病人情況描述(含劑量等) | Patient Situation (eg. dosage) |
| mechanism | 機制 | Mechanism |
| implication | 臨床處置建議(implication) | Implication |
| source_title | 原始文獻篇名 | Source Title |
| source_author | 原始文獻作者 | Source Author |
| source_citation | 原始文獻期刊名含頁碼 | Source Citation |
| source_url | 原始文獻網址連結 | Source URL |

Web Page Design

JavaScript is a front-end language used to create web pages. JavaScript was previously known as LiveScript and subsequently renamed JavaScript after collaboration with Netscape and Sun and inspiration by Java. JavaScript was renamed because the code is similar in appearance to Java, and there are some structural similarities. However, JavaScript and Java are not related.

Node.js [[4](#_ENREF_4)] is a high-performing, easily scalable platform that allows JavaScript to run [[5](#_ENREF_5)]. Node.js is created with scalability and instant response under a framework with ultra-large-scale data requests. A single-thread, asynchronous I/O, and event-driven programming design model was used to allow developers to easily develop highly scalable network services. There is no need for excessive complicated adjustments and program modifications to meet the network service requirements during different stages of development.

Node.js uses a Google Chrome Project V8 JavaScript Engine [[6](#_ENREF_6)] (available online: https://code.google.com/p/v8/). Node.js is extremely efficient, and its execution speed is quickly approaching that of native programs. JavaScript can also be applied to the development of back-end service programs, resulting in excellent performance [[1](#_ENREF_1)].

Over the past few years, the front-end web development industry has experienced rapid growth, and there are many excellent choices for front-end frameworks. Vue.js focuses on web technologies and is used to develop front-end frameworks for web interfaces. Vue.js is a very lightweight tool with responsive programming and componentization features. Furthermore, Vue.js can easily be combined with other frameworks and libraries. When using Vue.js, there is no need to find the DOM to access the data. Vue.js binds the DOM to the data, and developers only need to focus on the changes in the data. The largest difference between Vue.js and other traditional implementations of MVC JavaScript Frameworks, such as Angular.js and React.js, is that Vue.js can serve as a purely static front-end page that does not require php. Therefore, search results are obtained more quickly; it is an implementation of the MVVM architectural pattern [[7](#_ENREF_7)]. MVVM stands for Model-View-ViewModel. The “View” is the content seen by users. The “Model” is composed of the materials. The “View” binds the materials to the “ViewModel” and then uses the ViewModel to access this information in the future. The “ViewModel” is an object that links the view and the model. The main purpose is to allow web pages to dynamically load data rather than write content directly in the source code for traditional static pages. This renders updating much easier because the content can be changed according to the time, environment and/or results of the database operations without changing the source code. Vue.js mainly performs the work of the ViewModel, allows the View to bind and then automatically reacts to the View as the Model changes.

1. Guo J: Google's Webpage Language Node.js: Jiakui Consulting; 2015.

2. Node.js Domain

3. Sun H: Master Git Github Git Server in 24: Qifeng Consulting; 2017.

4. Pereira C.R. Introduction to Node.js. In: Building APIs with Node.js. Apress, Berkeley, CA; 2016.

5. Vinoski STaS: Node.js: Using JavaScript to Build High-Performance Network Programs. *IEEE Internet Computing* 2010, 14, no. 6,:80-83.

6. V8 JavaScript Engine. available online: https://code.google.com/p/v8/ [https://code.google.com/p/v8/]

7. Chen LY: Vue.js Front End Development. In: *People's Post Press.* 2017.
